# Interference of chemical defence and sexual communication can shape the evolution of chemical signals

**DOI:** 10.1038/s41598-017-18376-w

**Published:** 2018-01-10

**Authors:** Lisa Pfeiffer, Joachim Ruther, John Hofferberth, Johannes Stökl

**Affiliations:** 1 Institute of Zoology, University of Regensburg, Universitätsstraße 31, 93053 Regensburg, Germany; 2 Department of Chemistry, Kenyon College, 312 Tomsich Hall, Gambier, OH 43022 USA; 3 Present Address: Institute of Insect Biotechnology, Justus-Liebig-University Gießen, Heinrich-Buff-Ring 26-32, 35392 Gießen, Germany

## Abstract

According to current evolutionary theory, insect pheromones can originate from extant precursor compounds being selected for information transfer. This is exemplified by females of the parasitoid wasp *Leptopilina heterotoma* whose defensive secretion consisting mainly of (−)-iridomyrmecin has evolved secondary functions as cue to avoid other females during host search and as female sex pheromone. To promote our understanding of pheromone evolution from defensive secretions we studied the chemical ecology of *Leptopilina clavipes*. We show here that *L*. *clavipes* also produces a defensive secretion that contains (−)-iridomyrmecin as major component and that females use it to detect and avoid host patches occupied by other females. However, the female sex pheromone of *L*. *clavipes* consists solely of cuticular hydrocarbons (CHCs) and males did not respond to female CHCs if presented in combination with the defensive secretion containing (−)-iridomyrmecin. This is in contrast to other species of *Leptopilina*, in which the iridoid compounds have no inhibiting effect or even function as sex pheromone triggering courtship behaviour. This indicates that *Leptopilina* species differ in the cost-benefit ratio for males searching for females, which might explain the strong divergence in the composition of the sex pheromone in the genus.

## Introduction

Information transfer via chemical compounds is the most ancient and widespread form of communication and is found in bacteria, fungi, plants, and the animal kingdom. Although chemical communication has been thoroughly studied for several decades, the origin and the evolution of chemical signals is not well understood.

One hypothesis regarding the evolution of chemical signals is the so-called precursor hypothesis^1–4^. According to this hypothesis, any compound that is released by one individual and detected by another individual of the same species can acquire a communicative function and evolve into a chemical signal. In this way, for example, a hormone excreted with urine, a compound present on the cuticle of an insect to prevent desiccation, or a constituent of a defensive secretion released upon a predatory attack may serve as the starting point for the evolution of a pheromone. Other individuals of the same species may be able to detect the compound and use the information to their own advantage. For example, they might learn of the presence of the releasing individual and show a response. If this response benefits both the releasing and the receiving individual, chemical ritualization of information transfer via this compound may result in it becoming a true chemical signal^1–3^. Prime examples of this evolutionary process are the female sex pheromones of the goldfish and the Atlantic Salmon which have evolved from steroid and prostaglandin hormones contained in the urine of females^5^.

In addition to hormones, defensive compounds can become the precursors of pheromones. For many arthropods chemical defence is the most effective countermeasure against a predatory attack by other arthropods and against much larger predators such as birds, reptiles and mammals. More than fifty percent of all terrestrial arthropod orders contain species which use some kind of chemical deterrent^6^. Indeed, arthropods manifest an extraordinarily rich diversity of chemical defensive systems, including internal toxins, venoms, reflex bleeding, anal and oral discharges and glandular secretions^7^. Chemical compounds used in the interaction between individuals of different species and which only benefit the emitting individual are termed allomones^1^. As allomones are released into the environment in relatively large amounts and because they often can be detected by an unspecialized sensory system, they fulfil all prerequisites to act as precursors for the evolution of chemical communication according to the precursor hypothesis^1,3^.

The parasitoid wasp *Leptopilina heterotoma* is one of the best-investigated examples for the evolution of a defensive compound into a sex pheromone. Wasps of the genus *Leptopilina* parasitize the larvae of *Drosophila*, including *D*. *melanogaster* ^8,9^. Females of *L*. *heterotoma* produce a defensive secretion consisting of five iridoid compounds, with (−)-iridomyrmecin making up more than 80% of the secretion^10^. This secretion is produced and stored in mandibular glands and is used as defensive allomone against insect predators such as ants^10,11^. Females release the secretion when under attack and adjust the amount released based on the size of the predator^10,12^. When not under attack, females release very small amounts of the defensive secretion. The same secretion, although in much smaller quantities, serves as the female sex pheromone and attracts males and triggers courtship behaviour^13^. Females additionally use (−)-iridomyrmecin released by other females as competition avoidance cue to recognize already exploited host patches^13^. The threefold use of (−)-iridomyrmecin by *L*. *heterotoma* illustrates the evolutionary route from a defensive compound via a competition avoidance cue to a female sex pheromone. The congeneric species *L*. *boulardi* and *L*. *victoriae* also produce a defensive secretion containing iridomyrmecin and females of both species also use the iridoid compounds to avoid other females. But only in *L*. *boulardi* have the iridoid compounds from the defensive secretion also become a part of the sex pheromone, while the sex pheromone of *L*. *victoriae* consists of only CHCs.

It is surprising that *L*. *heterotoma* and *L*. *boulardi* use the defensive secretion in their sex pheromone, because a defensive compound with repellent or toxic properties should not evolve to attract other individuals of the same species when the detection of the compound indicates that the releasing individual is under attack, and there is a risk of harm to the attracted individual, either by the compound itself or by the predator attacking the releasing individual. One would expect that chemical defence mechanisms come with major costs, rendering an individual unattractive to mates or other conspecifics. Although trade-offs play a central role in evolutionary theory, the trade-off between two conflicting behavioural functions in the evolution of chemical communication is so far not well understood.

To better understand the evolution of sex pheromones from defensive secretions we studied the chemical ecology of the congeneric species *L*. *clavipes* by analysing the composition of its defensive secretion and by testing the role of the defensive compounds in the females’ host patch choice and sex pheromone. We show that the defensive secretion of *L*. *clavipes* consists mainly of (−)-iridomyrmecin and that females avoid host patches supplemented with (−)-iridomyrmecin or its epimer (+)-isoiridomyrmecin. The female sex pheromone of *L*. *clavipes* consists of cuticular hydrocarbons, which elicit courtship in males. However, males did not react to female CHCs if presented in combination with the defensive secretion. This is in contrast to other species of *Leptopilina*, in which the defensive secretion has no effect on the males’ courtship behaviour. Taken together these observations indicate that important differences in the cost-benefit ratios exist among *Leptopilina* species with respect to searching for mates by males and the use of the chemical defence by females.

## Results

### Chemical analysis

We identified iridoid compounds and CHCs in the extracts of females and males of *L*. *clavipes*. Females produced on average 259 ng (SD 93) of iridoid compounds, with (−)-iridomyrmecin being the major iridoid, making up 84% (217 ng) of the iridoid compounds. In addition to (−)-iridomyrmecin, females produced 7 other iridoid compounds. Except for (+)-isoiridomyrmecin, all iridoid compounds found in females were also found in males, but in much lower quantities (mean 127 ng, SD 40).

**Figure 1:**
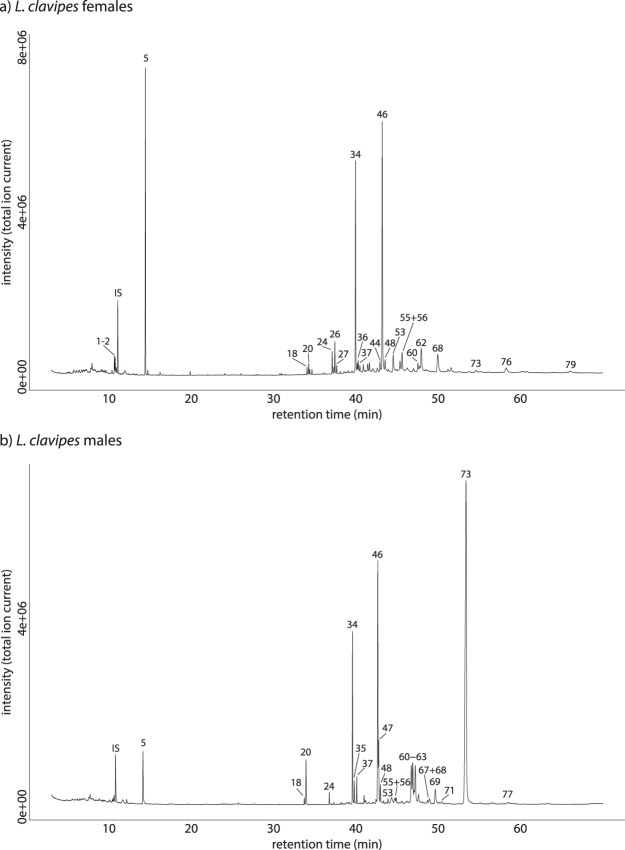
Total ion current chromatograms (TIC) of an extract of *L*. *clavipes* (**a**) females and (**b**) males. Numbers above peaks correspond to Table 1. Only peaks representing more than 0.5% of the total peak area of all compounds are indicated. IS – internal standard (5 ng methyl decanoate).

In total we identified 73 cuticular hydrocarbons in the extracts of female and male wasps (Table 1). Those were mainly methyl branched alkanes, but alkenes and alkadienes were also found in significant amounts. The CHC profile of males was dominated by 9,19-pentatriacontadiene (more than 40% of the CHCs) and 4-methyl alkanes with a chain length of 28 and 30 (Table 1, Fig. 1). The CHC profile of females was qualitatively and quantitatively similar, except for 9,19-pentatriacontadiene, which is produced only in traces by the females.

**Table 1:** Compounds identified in the extracts of females and males of *Leptopilina clavipes*. KRI = Kovats retention index on a non-polar (DPX-5) GC column. Diagn. Ion = diagnostic ions used in the identification of the compound. Diag. Ion DMDS = diagnostic ions of unsaturated compounds after derivatisation with DMDS. The percentage of compounds is based on the total peak area of all identified peaks. tr = trace amounts. Numbers of compounds correspond to Fig. 1.

No.	Compound	KRI	Diagn. Ions	Diagn. Ions DMDS	Females Mean %	Females SD %	Males Mean %	Males SD %
1	Iridodial 1	1313	168 (M+), 111, 135		1.07	0.52	0.13	0.13
2	Iridodial 2	1317	168 (M+), 109, 135		0.75	0.37	0.12	0.11
3	unknown, identical to P3 in^13^	1322	67, 81, 109, 152		0.51	0.23	0.24	0.14
4	unknown, identical to P4 in^13^	1365	67, 81, 109		0.38	0.28	0.22	0.28
5	(−)-iridomyrmecin	1467	168 (M+), 95, 109		14.81	5.65	2.89	1.31
6	(+)-isoiridomyrmecin	1479	168 (M+), 95, 109		0.23	0.09	tr	
7	Heptadecane	1700	240 (M+)		0.19	0.07	0.04	0.02
8	Octadecane	1800	254 (M+)		0.06	0.03	0.04	0.02
9	Nonadecane	1900	268 (M+)		0.10	0.04	0.05	0.03
10	Heneicosane	2000	282 (M+)		0.10	0.03	0.07	0.03
11	Eicosane	2100	296 (M+)		0.08	0.02	0.06	0.05
12	Docosane	2200	310 (M+)		0.03	0.01	0.03	0.04
13	4-methyl docosane	2262	324 (M+), 309 (M-15), 281		0.36	0.32	0.06	0.06
14	9-tricosene	2275	322 (M+), 97	416 (M+), 173, 243	0.30	0.23	0.04	0.03
15	7-tricosene	2282	322 (M+), 97	416 (M+), 145, 271	0.09	0.07		
16	Tricosane	2300	324 (M+)		0.10	0.06	0.05	0.05
17	Tetracosane	2400	338 (M+)		0.05	0.03		
18	4-methyl tetracosane	2463	352 (M+), 337 (M-15), 309		1.20	0.83	0.74	0.51
19	x,x-pentacosadiene^1^	2473	348 (M+), 96		0.23	0.16	0.12	0.05
20	9-pentacosene	2476	350 (M+), 97	444 (M+), 173, 271	1.13	0.67	1.86	0.64
21	7-pentacosene	2483	350 (M+), 97	444 (M+), 145, 299	0.48	0.27		
22	Pentacosane	2500	352 (M+)		0.56	0.37	0.07	0.04
23	Hexacosane	2600	366 (M+)		0.19	0.14	tr	
24	4-methyl hexacosane	2663	380 (M+), 365(M-15), 337		2.26	1.31	0.64	0.33
25	9-heptacosene	2677	378 (M+), 97	472 (M+), 173, 299	0.99	0.63	tr	
26	7-heptacosene	2685	378 (M+), 97	472 (M+), 145, 327	3.91	2.32	0.12	0.07
27	Heptacosane	2700	380 (M+)		1.09	0.74	tr	
28	13-methyl heptacosane and 11-methyl heptacosane	2730	379 (M-15), 196/197, 224/225 and 379 (M-15), 168/169, 252/253		0.22	0.08	0.03	0.01
29	5-methyl heptacosane	2748	379 (M-15), 337, 85		0.03	0.02	0.09	0.02
30	4-methyl heptacosane	2763	394 (M+), 379 (M-15), 351		0.17	0.05	0.12	0.07
31	Octacosane	2800	394 (M+)		0.40	0.07	0.18	0.16
32	x-methyl x-octacosene^1^	2832	406 (M+), 97		0.28	0.11	0.12	0.07
33	x,x-nonacosadiene^1^	2854	404 (M+), 96		0.37	0.21		
34	4-methyl octocosane	2862	408 (M+), 393 (M-15), 365		11.67	1.54	7.55	1.15
35	9-nonacosene	2877	406 (M+), 97	500 (M+), 173, 327	1.00	0.50	0.58	0.43
36	7-nonacosene	2886	406 (M+), 97	500 (M+), 145, 355	1.71	1.01	tr	
37	Nonacosane	2900	408 (M+)		1.02	0.55	1.07	1.23
38	15-methyl nonacosane and 13-methyl nonacosane	2926	407 (M-15), 224/225 & 407 (M-15), 196/197, 252/253		0.76	0.16	0.10	0.04
39	5-methyl nonacosane	2946	422 (M+), 407 (M-15), 85, 365		0.19	0.04	0.06	0.02
40	4-methyl nonacosane	2960	422 (M+), 407 (M-15), 379		0.68	0.12	0.44	0.15
41	3-methyl nonacosane and 5,x-dimethyl nonacosane^1^	2973	422 (M+), 407 (M-15), 393 & 421 (M-15), 379		0.91	0.28	0.28	0.14
42	Triacontane	3000	423(M+)		0.30	0.16	0.09	0.06
43	x-methyl x-triacontene^1^	3028	434 (M+)		0.84	0.26	0.24	0.13
44	x,x-hentriacosadiene^1^	3045	432 (M+), 96		1.85	0.60	0.19	0.12
45	x,x-hentriacosadiene^1^	3053	432 (M+), 96		0.23	0.10	tr	
46	4-methyl triacontane	3060	436 (M+), 421 (M-15), 393		19.63	2.40	14.25	5.08
47	15-hentriacontene & 14-hentriacontene	3064	434 (M+), 97	528 (M+), 257, 271 & 528 (M+), 243, 285			3.09	1.92
48	9-hentriacontene	3078	434 (M+), 97	528 (M+), 173, 355	1.87	0.86	0.69	0.37
49	x-hentriacontene^1^ (probably 7-hentriacontene)	3087	434 (M+), 97		0.23	0.13		
50	Hentriacontane	3100	436 (M+)		0.54	0.34	0.21	0.10
51	unknown	3110					0.05	0.04
52	unknown	3117					0.12	0.08
53	15-methyl hentriacontane & 13-methyl hentriacontane	3125	435 (M-15), 224/225, 252/253 & 435 (M-15) 196/197, 280/281		2.69	1.08	0.79	0.35
54	5-methyl hentriacontane	3145	435 (M-15), 85, 393		0.23	0.10	0.24	0.12
55	7,15-dimethyl hentriacontane	3161	449 (M-15), 435 (M-30), 112/113, 252/253, 239, 379		1.25	0.42	0.72	0.35
56	5,17-dimethyl hentriacontane	3172	449 (M-15), 435 (M-30), 84/85, 224/225, 267, 407		2.32	1.16	0.84	0.58
57	unknown	3191			0.15	0.07		
58	Dotriacontane	3200	450 (M+)		0.38	0.25	0.18	0.11
59	x-methyl x-dotriacontene^1^	3227	462 (M+)		0.68	0.23	0.32	0.20
60	x,x-tritriacontadiene^1^	3245	460 (M+), 96		2.43	0.59	2.74	0.74
61	x,x-tritriacontadiene^1^	3253	460 (M+), 96		0.76	0.14	3.48	1.07
62	4-methyl dotriacontane	3259	464 (M+), 449 (M-15), 421		3.10	0.39	1.50	0.60
63	16-tritriacontene		462 (M+), 97	556 (M+), 271, 285			2.46	0.66
64	x-tritriacontene^1^ (probably 9-tritriacontene)	3279	462 (M+), 97		0.78	0.43	0.41	0.11
65	x-tritriacontene^1^ (probably 7-tritriacontene)	3287	462 (M+), 97		0.19	0.10	tr	
66	Tritriacontane	3300	464 (M+)		0.12	0.15		
67	x-methyl x-tetratriacontene^1^	3315	476 (M+), 97	570 (M+), 131, 439 (prob. 6-en)			0.70	0.36
68	17-methyl tritriacontane & 15-methyl tritriacontane & 13-methyl tritriacontane	3324	463 (M-15), 252/253 & 463 (M-15), 224/225, 280/281 & 463 (M-15), 196/197, 308/309		3.83	1.33	1.36	0.68
69	x,x-tetratriacontadiene^1^	3343	474 (M+), 96		tr		1.45	0.23
70	x,x-dimethyl tritriacontane^1^	3357	57, 71, 85		tr		0.28	0.17
71	5,x-dimethyl tritriacontane^1^	3367	478 (M-15), 57, 71, 85, 436		tr		0.58	0.39
72	Tetratriacontane	3400	478 (M+)		tr		0.10	0.06
73	9,19-pentatriacontadiene	3443	488 (M+), 96	676 (M+), 173, 271, 311, 357, 409, 455, 535, 582	1.24	0.27	43.19	4.76
74	x-pentatriacontene^1^	3480	490 (M+), 97		0.43	0.17		
75	x-methyl x-pentatriacontene^1^	3515	57, 97, 111				0.33	0.19
76	15-methyl pentatriacontane	3521	491 (M-15), 224/225, 308/309		2.05	0.64	0.49	0.28
77	7,15-dimethyl pentatriacontane	3558	506 (M-15), 112/113, 239, 308/309, 435		tr		0.54	0.21
78	5,17-dimethyl pentatriacontane	3569	506 (M-15), 84/85, 239, 308/309, 464		tr		0.30	0.20
79	x,x-heptatriacontadiene^1^	3653	516 (M+), 96		1.22	0.52		

^1^The position of the double bond(s) and/or the methyl group(s) could not be determined.

### Female sex pheromone

**Figure 2:**
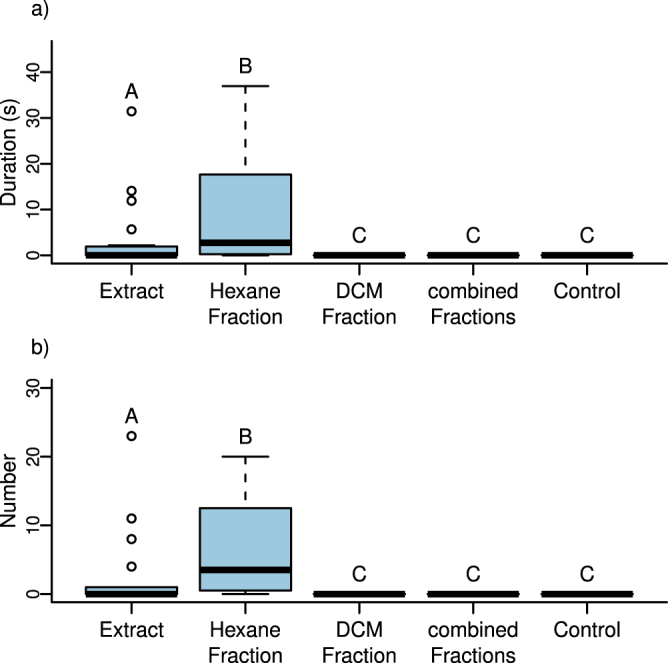
(**a**) Total duration of wing fanning behaviour and (**b**) number of wing fanning events shown by naïve virgin males of *L*. *clavipes* towards filter paper impregnated with the whole body extract of *L*. *clavipes* females, the hexane and DCM fractions thereof, the combined fractions, and the solvent control. Different letters indicate a significant difference (Kruskal Wallis ANOVA followed by pairwise Mann-Whitney U-Tests with Bonferroni-Holm correction, P < 0.05). For each experiment n = 20.

Males of *L*. *clavipes* showed very little wing fanning behaviour, in terms of both the number of wing fanning events and the duration of each event, when exposed to filter paper impregnated with the crude extract of females (containing CHCs and iridoids). Males showed significantly more wing fanning towards a filter paper impregnated with the hexane fraction of the female extract (containing only CHCs), but not towards a filter paper impregnated with the dichloromethane (DCM) fraction of the female extract (containing only iridoids) or the re-combined hexane and DCM fractions (Fig. 2a,b).

### Host patch choice

**Figure 3:**
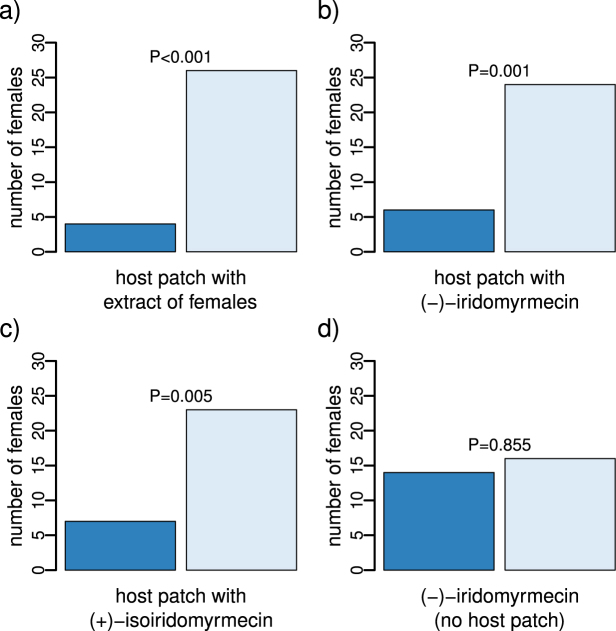
Frequency of decision for sample or control of mated *L*. *clavipes* females in a y-tube experiment when choosing between the odour of (**a**) an unexploited host patch and a host patch with extract of *L*. *clavipes* females, (**b**) an unexploited host patch and a host patch with synthetic (−)-iridomyrmecin, (**c**) an unexploited host patch and a host patch with synthetic (+)-isoiridomyrmecin, and (**d**) synthetic (−)-iridomyrmecin and the solvent control. Bar colours indicate sample (dark blue) and control (unexploited host patch or the solvent, light blue). P-values are given for the two-sided binomial test. For each experiment n = 30.

Females of *L*. *clavipes* searching for hosts avoided the odour of host patches to which an extract of females had been added and preferred the odour of host patch without female extract (Fig. 3a). The odour of the host patch was also avoided, if it was supplemented with synthetic (−)-iridomyrmecin or synthetic (+)-isoiridomyrmecin (Fig. 3b,c). (−)-iridomyrmecin itself, without the background odour of a host patch, had neither an attractive nor a repellent effect on females (Fig. 3d).

## Discussion

In this study, we show that the defensive compounds produced by females of the parasitoid wasp *L*. *clavipes* obstruct male attractiveness to females. In behavioural experiments, males did not show courtship behaviour to any extract or fraction thereof containing iridoid compounds. Only the hexane fraction of the female extract, which exclusively contained CHCs, was attractive to males of *L*. *clavipes*, while the combination of CHCs and iridoid compounds did not trigger courtship behaviour. Living females of *L*. *clavipes* are also attractive to males, because *Leptopilina* females store the iridoid compounds in mandibular glands and release them when under attack^10,11^. Therefore, living females of *L*. *clavipes* that are not acting in defense release only trace amounts of the iridoid compounds, which do not influence the response of males to CHCs.

*Leptopilina clavipes* is one of the few examples in which two classes of semiochemicals produced by the same individual can interfere and lead to a trade-off between the two important functions of those semiochemicals. Our data suggest that the use of a defensive allomone for protective purposes might reduce the chance of attracting potential mates. One of the few other examples of this phenomenon has been reported in the staphylinid beetle *Aleochara curtula*, where compounds from the defensive secretion (4-tridecene, dodecanal, 5-tetradecenal) inhibit male copulatory behaviour^14^. Interestingly, in the same beetle, a low concentration of the same compounds work synergistically with cuticular lipids and stimulate copulations in males^14^.

Our results show that the defensive secretion of *L*. *clavipes* consists of the same or very similar iridoid compounds as the defensive secretion of *L*. *heterotoma*, *L*. *boulardi* and *L*. *victoriae* ^10,13,15^. We could furthermore show that *L*. *clavipes* females, like females of *L*. *heterotoma* ^13^, also use iridomyrmecin to detect and avoid already exploited host patches. Therefore, these four *Leptopilina* species have a very similar chemical ecology in terms of the composition of the defensive secretion and the avoidance of iridomyrmecin by females during host patch choice.

**Figure 4:**
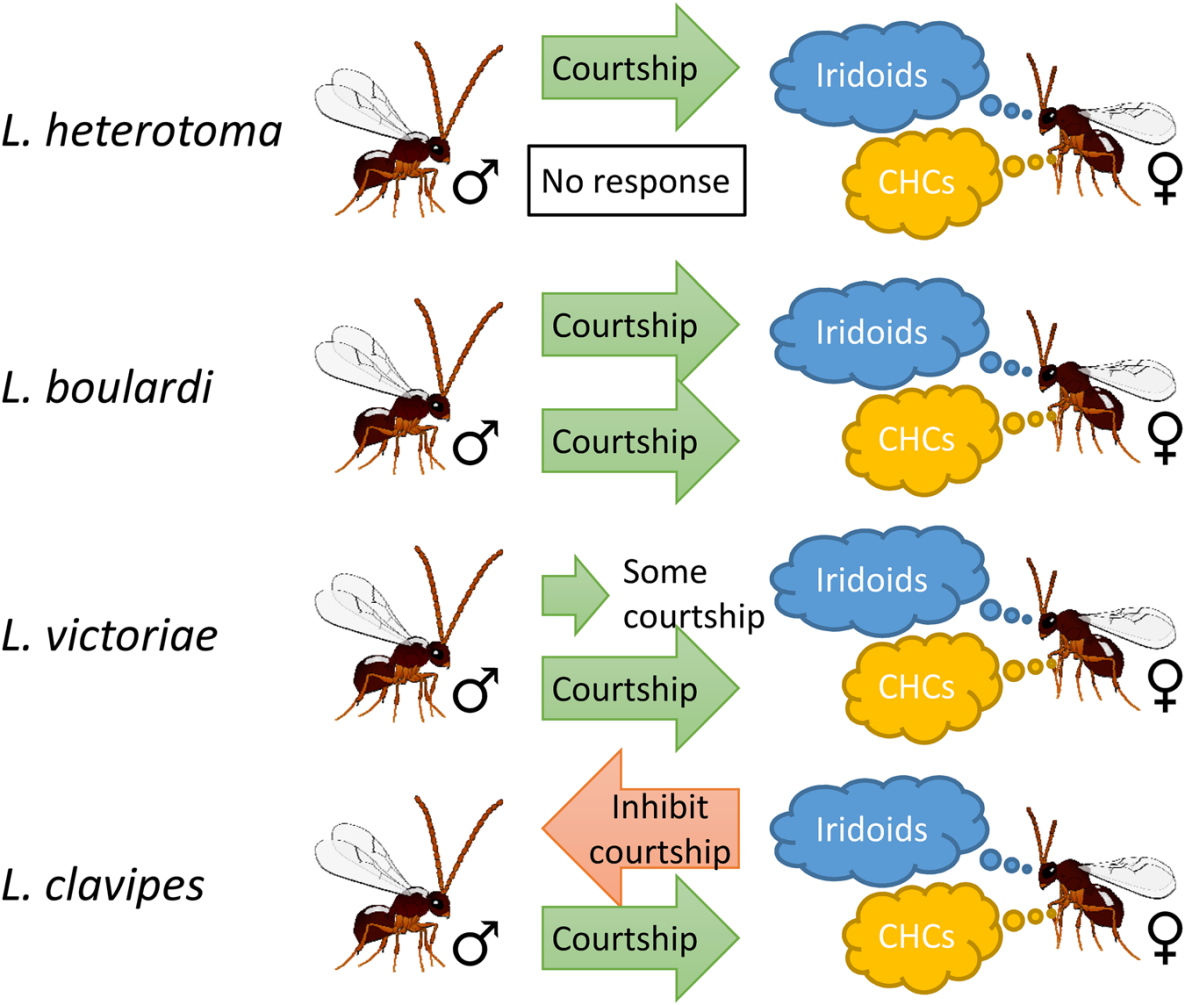
Illustration summarizing the response of males *of L*. *heterotoma*, *L*. *boulardi*, *L*. *victoriae* and *L*. *clavipes* to the defensive secretion (consisting of iridoid compounds) and the cuticular hydrocarbons (CHCs) of conspecific females.

However, the same four species differ significantly in the composition of the female sex pheromone and the response of males towards the defensive chemicals and sex pheromone (Fig. 4). In *L*. *heterotoma* the defensive secretion has been co-opted to function as female sex pheromone, while the female sex pheromone of *L*. *boulardi* consists of a combination of iridoid compounds and cuticular hydrocarbons. In *L*. *victoriae* and *L*. *clavipes* the iridoid compounds from the defensive secretion are not part of the female sex pheromones, which solely consist of CHCs. The strong diversification of the female sex pheromone despite the ability of all species to produce both iridoid compounds and CHCs cannot be explained by the need of a species specific sex pheromone alone. Qualitative and quantitative variation within the CHCs would allow for hundreds of different chemical profiles and distinct sex pheromone blends. Moreover, hundreds of different chemical blends based on iridoid compounds alone could have evolved. The variation of the female sex pheromone in *Leptopilina* might therefore be linked to the reaction of the males to the defensive secretion. Males of *L*. *heterotoma*, *L*. *boulardi*, and *L*. *victoriae* are not repelled by the iridoid compounds produced by females, irrespective of the composition of the female sex pheromone (Fig. 4). Males of *L*. *victoriae*, for example, show equal duration of wing fanning towards the CHCs as to a combination of CHCs and iridoid compounds^15^. In contrast, males of *L*. *clavipes* react with wing fanning to the females’ CHCs, but do not show courtship behaviour towards extracts containing CHCs and iridoids (Fig. 2). The different strategies of the males indicate differences in the cost-benefit ratios between species. On one hand, males benefit from being attracted towards the iridoid compounds by increasing the possibility to find a female. Iridoid compounds are more volatile than most CHCs produced by insects and therefore the male would be able to locate a female from a greater distance using iridoid compounds compared to CHCs. On the other hand, males attracted by the defensive secretion experience a higher risk of predation, as the predator triggering the release of the defensive secretion might still be present. The ratio of these costs and benefits likely determines the selective pressure on the males and consequently on the route in the evolution of the female sex pheromone.

We therefore see two contradicting forces in the evolution of pheromones from defensive compounds. On one hand, defensive compounds are very good candidates for the evolution of pheromone communication for three reasons. First, they are produced and released in relatively large amounts by the insect. Second, they are often volatile and can therefore be detected from a distance; and third, they can often be perceived by generalist odorant receptors.

On the other hand, most defensive compounds are repellent, because they are irritating or toxic. The receiving individual could therefore be directly harmed by the defensive compound. Furthermore, defensive compounds not only indicate the presence of the releasing individual but also the presence of a predator or threat. Using defensive compounds to locate females might lead to a trade-off for males between predation risk and reproduction. Therefore, in species with a high predation risk defensive secretion are not expected to evolve into sex pheromones. Unfortunately, measuring the predation risk, the frequency of the use of the defensive secretion, and the availability of females in natural populations of *Leptopilina* wasps is a challenging task.

Such a trade-off has rarely been observed in insects, but in vertebrates several such cases have been described. Males of the red-spotted newt, for example, are attracted by female pheromones but avoid conspecific alarm substances. If female pheromones are paired with alarm substances, males show an intermediate attraction^16^.

The costs for females using the defensive secretion also differs between *L*. *heterotoma* and *L*. *clavipes*. For females of *L*. *heterotoma* the use of the defensive secretion comes without costs in terms of reduced mate attraction. On the contrary, by releasing large quantities of defensive iridoids a female of *L*. *heterotoma* might even attract more potential mates. For females of *L*. *clavipes* the costs of using the defensive allomone vary with the frequency of its use. A high predator density might lead to an intense use of the chemical defence and a lower probability for the females to find a mate.

Our data highlight that closely related species with a very similar ecology can show large variation in their reaction towards defensive secretions released by the opposite sex, ranging from attraction to aversion, and that this variation can shape the evolution of chemical communication.

## Material and Methods

### Rearing of insects

We used *Drosophila virilis* as host to rear *Leptopilina clavipes*. *Drosophila virilis* was reared on a standard corn-based diet (500 ml water, 25 g sugar, 25 g cornmeal, 25 g wheat germ, 20 g baker’s yeast, 4 g agar, 2.5 ml propanoic acid) and kept at 25 °C, 60% humidity, and a 16:8 h light:dark cycle. About 30 flies (mixed sexes) were placed in a jar containing fresh fly food for oviposition. After 48 h the flies had laid a sufficient number of eggs and were removed from the jar and 5–10 mated *L*. *clavipes* females were put in the jar to parasitize the fly larvae. A few days before emergence of the wasps (approximately three weeks later) parasitized pupae were removed from the rearing jar and put singly into 1.5 ml microcentrifuge tubes to get naïve, virgin wasps of known age. Wasps were sexed using morphological characters (males have much longer antennae than females).

### Chemical analysis

To identify the compounds in the defensive secretion and the female sex pheromone we extracted female and male wasps for 10 min in 5 μl dichloromethane (DCM) per wasp. To disentangle the functions of iridoids and CHCs we fractioned the extract of females by solid phase extraction (SPE). Prior to SPE, the raw extract was dried under a stream of nitrogen, and the sample was redissolved in 50 μl hexane. Cyanopropyl-bonded silica gel columns (50 mg, DSC-CN, Sigma-Aldrich, Taufkirchen, Germany) were pre-conditioned by rinsing them with 2 ml each of DCM and hexane. The samples were applied to the column and eluted with 300 μl hexane followed by 300 μl DCM. Between elution with hexane and elution with DCM, the column was flushed with additional 300 μl hexane. The composition of both fractions was analysed by GC-MS (see below). The hexane fraction contained the CHCs and the DCM fraction contained the iridoids.

Extracts and fractions were analysed on a Shimadzu GC2010 gas chromatograph (GC) connected to a QP2010 plus mass spectrometer (MS; Shimadzu, Duisburg, Germany). The GC was equipped with a non-polar capillary column (BPX-5, 30 m length, 0.25 mm inner diameter, 0.25 μm film thickness; SGE Analytical Sciences, Milton Keynes, UK). Helium was used as carrier gas with a constant linear velocity of 50 cm s^−1^. Sample volumes of 1 μl were injected splitless at an injector temperature of 280 °C. The temperature programme of the GC oven started at 80 °C and was raised by 5 °C min^−1^ to 280 °C, where it was kept for 20 min. The MS was run in electron impact (EI) mode at 70 eV and set to a scan range from 35–600 *mz* ^−1^.

To quantify the compounds produced by males and females of *L*. *clavipes*, single individuals (for each sex N = 12) were extracted with 20 µl DCM containing 5 ng µl^−1^ methyl decanoate as internal standard and analysed as described above. Compounds were quantified by comparing the peak area of the compounds with that of the internal standard.

Iridoid compounds were identified by comparing the retention time and mass spectra of the compounds with those of authentic reference compounds (see ref.^13^ for synthesis method) and the compounds identified in the extracts of other species of the genus^10,13,15^. To separate the enantiomers of iridomyrmecin, the GC was equipped with a chiral ß-cyclodextrin column (BetaDEX 225, 30 m length, 0.25 mm inner diameter, 0.25 μm film thickness; Sigma-Aldrich, Taufkirchen, Germany). For enantioselective analyses the injector temperature was set to 200 °C. The temperature program of the GC oven started at 80 °C and increased by 6 °C min^−1^ to 200 °C. The final temperature was held for 20 min.

Saturated *n*-alkanes were identified by comparing their retention times and mass spectra with those of a reference mix of alkanes (Sigma-Aldrich). Methyl-branched alkanes were identified by comparing the Kovats retention indices of the peaks with data from the literature^17^ and by the diagnostic ions resulting from the favoured fragmentation at the branching points^18^. The positions of double bonds in unsaturated hydrocarbons were determined by the diagnostic ions of the compounds after derivatisation with dimethyl disulphide^19^. Derivatised samples were analysed on the same GC-MS system as the underivatised samples, but with a higher final oven temperature (300 °C instead of 280 °C) and an increased mass range (35–800 *mz* ^−1^).

### Female sex pheromone

We used the duration of the wing fanning behaviour shown by males during courtship to assess the attractiveness of extracts and fractions. For this, we applied 2 µl of the extract, fractions thereof (in both cases equivalent to one 10^th^ of a female) or the pure solvent onto small discs of filter paper (5 mm diameter). The solvent was allowed to evaporate (approx. 30 sec) and the filter paper was placed in the centre of a small arena (15 mm diameter, 2 mm high, bottom made of glass). A single virgin and naive male (1 to 3 days old) was carefully introduced to the arena, which was then covered with a glass lid. The behaviour of the male was observed and recorded as digital video for 2 min. The number of the wing fanning events and their total duration was determined by analysing the video files with the software “The Observer” (Noldus Information Technology, Wageningen, The Netherlands). After each experiment, the arena was rinsed with ethanol and left to dry for 2 min. Experiments were conducted at room temperature and repeated 20 times for each treatment.

### Host patch choice

We used a y-tube olfactometer to test whether females of *L*. *clavipes* use (−)-iridomyrmecin to avoid competition during host search as we have described previously for *L*. *heterotoma* ^13^. The y-tube was made of glass with an inner diameter of 1.5 cm. The base and arms had a length of 6 cm and 9 cm, respectively, and the arms were oriented at an angle of 45°. The y-tube was positioned at a 30° angle, with the two arms pointing up the slope, and was illuminated from above by two neon tubes (8 W). Humidified air was pumped into the arms at a combined rate of 150 ml min^−1^ via two Erlenmeyer flasks (50 ml, one for each arm). Each flask contained an artificial host patch consisting of 5 g of *Drosophila* rearing substrate, on which approx. 5 *D*. *virilis* females had been allowed to oviposit for 48 h. The test compounds (crude extract of females, synthetic (−)-iridomyrmecin, or (+)-isoiridomyrmecin, always equivalent to one 10^th^ of a female) were applied on discs of filter paper (5 mm diameter), which were placed directly into one of the arms. To test whether females avoid (−)-iridomyrmecin only in the context of host search, we performed a control experiment in which we removed the host patches and females could choose between (−)-iridomyrmecin and the solvent control. For each run of the experiment one 7–10 day-old mated *L*. *clavipes* female was released into the base of the y-tube. The test lasted for 10 min or until the individual passed a ‘decision line’, which was marked in each arm 2 cm beyond the branching point. After each run the y-tube was turned and treatment and control odour was swapped. After every second run the y-tube was rinsed with ethanol and hot water. To increase the number of responding females, the females to be tested were allowed to lay eggs for 1 h directly before the tests by giving them access to host larvae in the same type of host patch as used in the experiment. Each individual was used for only one test (n = 30 for each treatment).

### Statistical analysis

We tested for a significant difference in the number and the total duration of the wing fanning behaviour between treatments using a Kruskal-Wallis-ANOVA followed by pairwise Mann-Whitney U-tests, corrected for multiple comparison using the method of Bonferroni. The decisions of the females in the y-tube were tested using a two-sided binomial test. All statistics were done in R Version 3.3.0^20^.

### Data availability

All datasets generated during this study are available from the corresponding author on reasonable request.
